# Echocardiographic markers of atrial retroactivation during junctional rhythm

**DOI:** 10.1093/ehjci/jeae228

**Published:** 2024-08-27

**Authors:** Emanuele Maria Renga, Gianluca Massaro, Gaetano Idone, Emanuele Di Marco, Giuseppe Massimo Sangiorgi

**Affiliations:** Department of Cardiology, Tor Vergata Hospital of Rome, University of Rome “Tor Vergata”, Viale Oxford 81, 00133 Rome, Italy; Department of Cardiology, Tor Vergata Hospital of Rome, University of Rome “Tor Vergata”, Viale Oxford 81, 00133 Rome, Italy; Department of Cardiology, Tor Vergata Hospital of Rome, University of Rome “Tor Vergata”, Viale Oxford 81, 00133 Rome, Italy; Department of Cardiology, Tor Vergata Hospital of Rome, University of Rome “Tor Vergata”, Viale Oxford 81, 00133 Rome, Italy; Department of Cardiology, Tor Vergata Hospital of Rome, University of Rome “Tor Vergata”, Viale Oxford 81, 00133 Rome, Italy; Department of Biomedicine and Prevention, University of Rome ‘Tor Vergata’, 00133 Rome, Italy

A 75-year-old man presented to the Emergency Department with dyspnoea and discomfort. The 12-lead electrocardiogram (ECG) reveals a junctional rhythm (JR) with a heart rate of 55 bpm (*Panel A*), accompanied by retrograde P waves (arrows). *Panel B* displays pulsed wave Doppler at the mitral valve (MV). Interestingly, early systole is characterized by reverse blood flow (white arrows), corresponding to the retrograde P-wave on the ECG. This atrial retroactivation during systole leads to partial MV opening or ineffective closure. During this phase, the higher intracavitary pressure in the left ventricle permits early systolic blood flow towards the left atrium.

**Figure:**
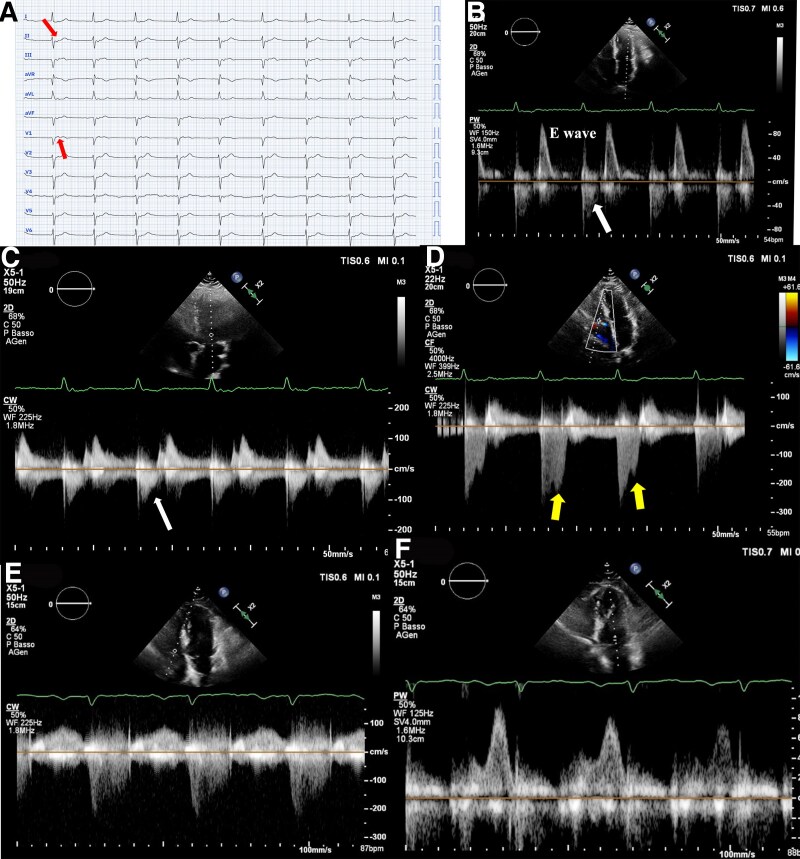


Mitral continuous wave (CW) Doppler (*Panel C*) shows a negative wave (white arrow) that synchronizes with the retrograde P-wave. *Panel D* presents tricuspid valve (TV) CW Doppler, where mild systolic tricuspid regurgitation (TR) is observed. Notably, a second regurgitant jet (arrows) occurs shortly after the retrograde atrial activation, in the later part of systole. This atrial retroactivation, due to the JR, prompts TV opening, and the pressure gradient between the right ventricle and right atrium causes the second jet. Interestingly, the TR jet resulting from atrial retroactivation is more pronounced than the mitral jet, likely due to the lower right ventricular pressure allowing a wider TV opening compared with the MV.

During sinus rhythm (SR), the secondary TR jet is absent (*Panel E*), and the blood flow across the MV (*Panel F*) exhibits characteristics typical of first-grade diastolic dysfunction, without a reverse A-wave. It is noteworthy that the E-wave velocity during JR is higher than during SR, possibly due to increased atrial filling from the additional regurgitant flow during JR, which subsequently enhances left ventricular filling during the rapid filling phase.

**Funding:** None declared.

**Data availability:** The data that support the findings of this study are available from the corresponding author, G.M., upon reasonable request.

